# Supplementary material for: Massive Open Online Course (MOOC) Opportunities in Health Education (HE) in a mandatory social isolation context
Source: F1000Res. 2022 May 26;10:322. Originally published 2021 Apr 26. [Version 2] doi: 10.12688/f1000research.52049.2 (PMC10915359; doi:10.12688/f1000research.52049.2)
Supplement: Peer review history

# Reviewer report: Reviewer response for version 2

**DOI:** 10.5256/f1000research.134018.r226861

**Published:** 2024-03-05

**Authors:** Khin Thandar Aung

**Affiliations:** 1 Kulliyyah of Nursing, International Islamic University, Kuala Lumpur, Malaysia

Regarding the specific systematic review paper, I would like to suggest the following to the authors:

1. The research topic and specific research question should be addressed in the review.
2. Methodology should be string such as the methods employed for the literature search, screening, and selection of studies as well as inclusion and exclusion criteria should be mentioned.
3. The PRISMA checklist and how the authors adhered to its reporting guidelines are suggested to apply for systematic literature review.
4. The specific quality appraisal tools used and the overall quality scores of included studies should be included in this review.
5. The synthesis and interpretation of findings, including potential limitations and future research directions are suggested.
6. There is no conclusion part written in the manuscript (please write a conclusion).

Is the work clearly and accurately presented and does it cite the current literature?

Partly

If applicable, is the statistical analysis and its interpretation appropriate?

Yes

Are all the source data underlying the results available to ensure full reproducibility?

Yes

Is the study design appropriate and is the work technically sound?

Yes

Are the conclusions drawn adequately supported by the results?

Yes

Are sufficient details of methods and analysis provided to allow replication by others?

Yes

Reviewer Expertise:

Public health emergencies, developing MOOC courses, Instructional design in education.

I confirm that I have read this submission and believe that I have an appropriate level of expertise to confirm that it is of an acceptable scientific standard.

# Reviewer report: Reviewer response for version 2

**DOI:** 10.5256/f1000research.134018.r139136

**Published:** 2022-09-05

**Authors:** Maya Adam

**Affiliations:** 1 Stanford University, Stanford, CA, 94305, USA

These changes look good to me. Best of luck to the authors and congratulations on an excellent addition to the literature.

 I approve without reservations.

Is the work clearly and accurately presented and does it cite the current literature?

Yes

If applicable, is the statistical analysis and its interpretation appropriate?

I cannot comment. A qualified statistician is required.

Are all the source data underlying the results available to ensure full reproducibility?

No source data required

Is the study design appropriate and is the work technically sound?

Yes

Are the conclusions drawn adequately supported by the results?

Yes

Are sufficient details of methods and analysis provided to allow replication by others?

Partly

Reviewer Expertise:

NA

I confirm that I have read this submission and believe that I have an appropriate level of expertise to confirm that it is of an acceptable scientific standard.

# Reviewer report: Reviewer response for version 1

**DOI:** 10.5256/f1000research.55275.r121485

**Published:** 2022-02-16

**Authors:** Maya Adam

**Affiliations:** 1 Stanford University, Stanford, CA, 94305, USA

In general, I think this is a very interesting manuscript and it makes a potentially valuable contribution to the literature on open online public health education.

 Some suggestions for improvement follow:

 In the background/rationale for the study, I wonder if the authors might also consider adding some brief statistics about the rise in global engagement with online courses that was spurred by the pandemic? Then the narrative would roughly go: 1) Routine care and preventive health visits went down, 2) General education through open online courses went up, 3) Can open online public health courses function as a potential bridge at times like these when face-to-face interactions are limited?

 In the methods section of the abstract, it would be helpful to briefly (but a bit more clearly) describe the inclusion and exclusion criteria for the courses described in this study.

 I think the conclusion in this study could be more clearly worded, especially in the abstract. Phrases like “in health promotion issues” and “multiply initiatives in distant territories” are somewhat vague and could be stated more clearly. I also think this study speaks to the potential for massive open online public health courses to increase access to important health promotion messages, even outside of “times of pandemic and compulsory social isolation”. This is an interesting direction for future research that could be stated here.

 I have to preface this next comment by applauding the authors for their ability to write an academic manuscript in a language that may not be their first, based on their institutional affiliations. I could not have written an academic manuscript like this in a second language. One comment that I hope will be helpful: In general, the use of strong, active verbs throughout would strengthen the writing stylistically. For example, a sentence like *“In December 2019, the COVID-19 pandemic was triggered by severe acute respiratory syndrome coronavirus 2 (SARSCov-2), revealing the fragility of the health systems of developing countries.” *Could be rewritten to read: “In December 2019, the severe acute respiratory syndrome coronavirus 2 (SARSCov-2) triggered the COVID-19 pandemic.” Another example “ *It has been estimated that maternal and child neglect in LMICs could be devastating…” *could be more strongly worded as “Researchers suggest that maternal and child health neglect…” (or similar).

 Secondly, longer “run-on” sentences can become sources of confusion. An example of this is your sentence: “ *The pandemic context requires changes and identification of strategies that can help meet the indirect effects of neglect of* *diseases not related to COVID-19 in health systems.”* Perhaps this sentence could be revised to be simpler and more direct so that it’s easier to understand on first pass.

 My final suggestion: it might be interesting to include information about enrollment number for the different courses. These are often available to the public and could give a better sense of the potential for global reach and scaling of these courses.

Is the work clearly and accurately presented and does it cite the current literature?

Yes

If applicable, is the statistical analysis and its interpretation appropriate?

I cannot comment. A qualified statistician is required.

Are all the source data underlying the results available to ensure full reproducibility?

No source data required

Is the study design appropriate and is the work technically sound?

Yes

Are the conclusions drawn adequately supported by the results?

Yes

Are sufficient details of methods and analysis provided to allow replication by others?

Partly

Reviewer Expertise:

Health communication, health promotion

I confirm that I have read this submission and believe that I have an appropriate level of expertise to confirm that it is of an acceptable scientific standard, however I have significant reservations, as outlined above.

# Response

**Published:** 2022-05-12

**Authors:** GANDY KERLIN GANDY KERLIN

**Affiliations:** Unidad de Conocimiento y Evidencia. Universidad Peruana Cayetano Heredia, Peru

Dear Maya Adam

 We are very grateful for your comments and suggestions. Below are the responses to each of the comments:

 In general, I think this is a fascinating manuscript and it makes a potentially valuable contribution to the literature on open online public health education.

 Some suggestions for improvement follow:

**First suggestion:**

 In the background/rationale for the study, I wonder if the authors might also consider adding some brief statistics about the rise in global engagement with online courses that was spurred by the pandemic? Then the narrative would roughly go: 1) Routine care and preventive health visits went down, 2) General education through open online courses went up, 3) Can open online public health courses function as a potential bridge at times like these when face-to-face interactions are limited?

**Answer:**

 Thank you very much for your comments; it’s very interesting. We have added statistics about online courses during the pandemic according to class central and Shravan Goli, boss of the products to MOOCs Coursera. Also, we have structured the introduction according to your suggestion.

**Second suggestion:**

 In the methods section of the abstract, it would be helpful to briefly (but a bit more clearly) describe the inclusion and exclusion criteria for the courses described in this study.

**Answer:**

 We consider this recommendation very precise. We briefly added the inclusion and exclusion criteria.

**Third suggestion:**

 I think the conclusion in this study could be more clearly worded, especially in the abstract. Phrases like “in health promotion issues” and “multiply initiatives in distant territories” are somewhat vague and could be stated more clearly. I also think this study speaks to the potential for massive open online public health courses to increase access to important health promotion messages, even outside of “times of pandemic and compulsory social isolation”. This is an interesting direction for future research that could be stated here.

**Answer:**

 Your comment is very pertinent. We have omitted some phrases and modified the conclusion in the abstract.

**Fourth suggestion**

 I have to preface this next comment by applauding the authors for their ability to write an academic manuscript in a language that may not be their first, based on their institutional affiliations. I could not have written an academic manuscript like this in a second language. One comment that I hope will be helpful: In general, the use of strong, active verbs throughout would strengthen the writing stylistically. For example, a sentence like “In December 2019, the COVID-19 pandemic was triggered by severe acute respiratory syndrome coronavirus 2 (SARSCov-2), revealing the fragility of the health systems of developing countries.” Could be rewritten to read: “In December 2019, the severe acute respiratory syndrome coronavirus 2 (SARSCov-2) triggered the COVID-19 pandemic.” Another example “It has been estimated that maternal and child neglect in LMICs could be devastating…” could be more strongly worded as “Researchers suggest that maternal and child health neglect…” (or similar).

 Secondly, longer “run-on” sentences can become sources of confusion. An example of this is your sentence: “The pandemic context requires changes and identification of strategies that can help meet the indirect effects of neglect of diseases not related to COVID-19 in health systems.” Perhaps this sentence could be revised to be simpler and more direct so that it’s easier to understand on first pass.

**Answer:**

 Thank you very much for your accurate comments and examples. We have identified different phrases longer and that can cause confusion. We have modified some sentences for a better understanding.

**Fifth suggestion:**

 My final suggestion: it might be interesting to include information about enrollment number for the different courses. These are often available to the public and could give a better sense of the potential for global reach and scaling of these courses.

**Answer:**

 Thank you very much for your comment. We are trying to keep up with the number of people enrolled, however most of these courses are currently closed and indicate future dates to start again. In other cases, they are in a new phase of registration for the course. So, it would be very difficult to know exactly the number of people enrolled in the course.

# Reviewer report: Reviewer response for version 1

**DOI:** 10.5256/f1000research.55275.r85974

**Published:** 2021-06-21

**Authors:** Waleed Al-Rahmi

**Affiliations:** 1 School of Education, Faculty of Social Sciences and Humanities, Universiti Teknologi Malaysia, Johor Bahru, Malaysia

1. The aim of this research not clear. The authors should clarify the aims.

 2. The research problems are not clear. Therefore, the authors should address the research gap first, and I suggest adding new section to explain about the real problems.

 3. I suggest adding research model, and test this model by Structural Equation Modeling (SEM), Why? Because we need to see the validation of theories applied in this research.

 4. I suggest for authors to read and cite the following references:

- A. The use of Massive Open Online Courses (MOOCs) in blended learning courses and the functional value perceived by students
- B. Integrating innovation diffusion theory with technology acceptance model: Supporting students’ attitude towards using a massive open online courses (MOOCs) systems.
- C. Massive Open Online Courses: enhancing caregiver education and support about dementia care towards and at end of life.
- D. Massive Open Online Courses (MOOCs): Data on Higher Education.
- E. Perceived user satisfaction and intention to use massive open online courses (MOOCs).
- F. Predicting user perceived satisfaction and reuse intentions toward Massive Open Online Courses (MOOCs) in the Covid-19 pandemic: An application of the UTAUT model and quality factors.

5.    The research methodology is not clear, the authors should explain more and add some references.  

 6.    The results analysis is not enough to show the research contributions. Therefore, the authors should add more analysis.

 7.    The authors should explain more what is the difference between this research with prior experimental results, and related research.

 8.    The authors should add new section about the limitations of this research, as well as what is the future research?

Is the work clearly and accurately presented and does it cite the current literature?

Partly

If applicable, is the statistical analysis and its interpretation appropriate?

No

Are all the source data underlying the results available to ensure full reproducibility?

No source data required

Is the study design appropriate and is the work technically sound?

Partly

Are the conclusions drawn adequately supported by the results?

Partly

Are sufficient details of methods and analysis provided to allow replication by others?

No

Reviewer Expertise:

computer and education

I confirm that I have read this submission and believe that I have an appropriate level of expertise to state that I do not consider it to be of an acceptable scientific standard, for reasons outlined above.

## References

[1] : The use of Massive Open Online Courses (MOOCs) in blended learning courses and the functional value perceived by students. *Computers & Education* .2021;161: 10.1016/j.compedu.2020.104077 10.1016/j.compedu.2020.104077

[2] : Integrating innovation diffusion theory with technology acceptance model: supporting students’ attitude towards using a massive open online courses (MOOCs) systems. *Interactive Learning Environments* .2019; 10.1080/10494820.2019.1629599 1-13 10.1080/10494820.2019.1629599

[3] : Massive Open Online Courses: enhancing caregiver education and support about dementia care towards and at end of life. *Age and Ageing* .2020;49(2) : 10.1093/ageing/afz150 171-174 10.1093/ageing/afz150 31782761

[4] : Massive Open Online Courses (MOOCs): Data on higher education. *Data Brief* .2019;22: 10.1016/j.dib.2018.11.139 118-125 10.1016/j.dib.2018.11.139 30581914 PMC6297852

[5] : Perceived user satisfaction and intention to use massive open online courses (MOOCs). *Journal of Computing in Higher Education* .2021;33(1) : 10.1007/s12528-020-09257-9 85-120 10.1007/s12528-020-09257-9

[6] : Predicting user perceived satisfaction and reuse intentions toward Massive Open Online Courses (MOOCs) in the Covid-19 pandemic. *International Journal of Research in Business and Social Science (2147- 4478)* .2021;10(2) : 10.20525/ijrbs.v10i2.1045 1-11 10.20525/ijrbs.v10i2.1045

# Response

**Published:** 2022-05-12

**Authors:** GANDY KERLIN GANDY KERLIN

**Affiliations:** Unidad de Conocimiento y Evidencia. Universidad Peruana Cayetano Heredia, Peru

Dear Waleed Mugahed Al-Rahmi

 We are grateful for your comments. Our responses are described below.

**1. The aim of this research not clear. The authors should clarify the aims**.

 Thank you for your comments. We had improved the description of the aims in the last paragraph.

**2. The research problems are not clear. Therefore, the authors should address the research gap first, and I suggest adding new section to explain about the real problems.**

 We are grateful for your comments, so we have modified the introduction, reworded the justification of the study and included some additional references.

**3. I suggest adding research model, and test this model by Structural Equation Modeling (SEM), Why? Because we need to see the validation of theories applied in this research.**

 Your comment is interesting and would certainly enhance research aimed at assessing causality itself. Structural equation modeling (SEM) is a multivariate statistical technique for testing and estimating causal relationships from statistical data and qualitative assumptions about causality. On the other hand, our study is of cross-sectional design and we set out from the beginning to perform descriptive analysis using secondary information. Therefore, the use of structural equation modeling (SEM) is not part of our methodology.

**4. I suggest for authors to read and cite the following references:**

 A. The use of Massive Open Online Courses (MOOCs) in blended learning courses and the functional value perceived by students

 B. Integrating innovation diffusion theory with technology acceptance model: Supporting students’ attitude towards using a massive open online courses (MOOCs) systems.

 C. Massive Open Online Courses: enhancing caregiver education and support about dementia care towards and at end of life.

 D. Massive Open Online Courses (MOOCs): Data on Higher Education.

 E. Perceived user satisfaction and intention to use massive open online courses (MOOCs).

 F. Predicting user perceived satisfaction and reuse intentions toward Massive Open Online Courses (MOOCs) in the Covid-19 pandemic: An application of the UTAUT model and quality factors.

 Thank you for sharing these studies with us. We visualize that the studies focus on measures of effect on participants participating in massive open online courses (MOOCs). Therefore, we believe that our study aims to identify and describe MOOCs that provide health education, promotion and prevention opportunities for the general population during a COVID-19 pandemic. In this opportunity, we did not set out to learn about the results in the participants of this study.

**5.    The research methodology is not clear, the authors should explain more and add some references.**

 Thank you for your comment, we would like to know what part of the methodology is not clear to you. We would be happy to make modifications to make it more understandable for you.

**6.    The results analysis is not enough to show the research contributions. Therefore, the authors should add more analysis.**

 Thank you very much for your appreciation. We value more complex analyses such as multivariate statistical analyses in research. However, in this opportunity and due to the context of the pandemic, we thought it was convenient to carry out a research that would allow us to demonstrate the offer of courses related to health education focused on prevention in a context where hospitals were not attending patients focused on disease prevention, given that the cases of COVID-19 were increasing.

**7.    The authors should explain more what is the difference between this research with prior experimental results, and related research.**

 Thank you for your comment. In the second paragraph of the discussion, we note many courses that are specialized and aimed at professionals. These courses were not included in our research as our objective is to describe the supply of courses available on health education (prevention and promotion), not disease treatment. We did not find previous research describing these types of courses on virtual platforms.

**8.    The authors should add new section about the limitations of this research, as well as what is the future research?**

 In the antepenultimate paragraph of the discussion, we describe our limitations for example the criteria for choosing a course were exclusively the decision of the researcher.  Regarding future research, in the last paragraph of the discussion, we stated that future research should aim at assessing the quality of the content of these courses, as our research does not address this.
